# Brca1 Is Upregulated by 5-Aza-CdR and Promotes DNA Repair and Cell Survival, and Inhibits Neurite Outgrowth in Rat Retinal Neurons

**DOI:** 10.3390/ijms19041214

**Published:** 2018-04-17

**Authors:** Qiyun Wang, Lijun Xu, Pei Chen, Zhuojun Xu, Jin Qiu, Jian Ge, Keming Yu, Jing Zhuang

**Affiliations:** State Key Laboratory of Ophthalmology, Zhongshan Ophthalmic Center, Sun Yat-sen University, Guangzhou 510060, China; wqiy@mail2.sysu.edu.cn (Q.W.); xlj727@163.com (L.X.); peichen912@163.com (P.C.); xuzhj7@mail2.sysu.edu.cn (Z.X.); qiujin19940916@163.com (J.Q.); gejian@mail.sysu.edu.cn (J.G.); yukeming@mail.sysu.edu.cn (K.Y.)

**Keywords:** Brca1, 5-Aza-CdR, neurite outgrowth, retina

## Abstract

Previous studies have reported that Brca1 acts as a “hinge” in the development of the central nervous system (CNS). However, the precise role of Brca1 in rat retinal neurons remains unclear. Here, we found that Brca1 is developmentally downregulated and silenced in adult retina. Brca1 was upregulated in rat primary retinal neurons by 5-Aza-2′-deoxycytidine (5-Aza-CdR) treatment. Moreover, the upregulation of Brca1 by both 5-Aza-CdR and transgenic Brca1 promoted genomic stability and improved cell viability following exposure to ionizing radiation (IR). Furthermore, transgenic Brca1 significantly inhibited neurite outgrowth of retinal neurons, which implicates that Brca1 silencing promotes cell differentiation and determines neuronal morphology. Taken together, our results reveal a biological function of Brca1 in retinal development.

## 1. Introduction

Breast cancer type 1 susceptibility protein (Brca1) was originally cloned as a human tumor suppressor, with mutations in the gene associated with an increased risk of developing breast and ovarian cancers [1,2]. Soon after, numerous studies demonstrated that Brca1 plays versatile roles in development and tumorigenesis. Brca1 is a nuclear–cytoplasmic shuttling protein which may be functionally regulated via active shuttling between cellular compartments [3]. Brca1 is well known to be involved in DNA repair and to play a central role in cellular growth control and genomic integrity [4,5,6,7]. Moreover, Brca1 is an important component of pathways that regulate ubiquitination and transcription [8].

Although studies of Brca1 in the central nervous system (CNS) are limited, there is strong evidence indicating that Brca1 may play an important role in neural development. Mice carrying a gene deletion for Brca1 show embryonic lethality and various degrees of abnormalities in the neuroepithelium and neural tube [9,10]. Separately, the expression of Brca1 has been detected in the neuroepithelium of the embryonic rat brain. Over development, the expression of Brca1 gradually declines and in many cases is silenced by the adult stage in all brain regions except for the walls of the ventricles and the choroid plexus, which consist of neural stem cells. Kondo et al. also reported that Brca1 is not detectably expressed in oligodendrocyte precursor cells (OPCs), but becomes re-expressed in neural stem-like cells (NSLCs) which derive from the de-differentiation of OPCs in vitro [11]. Moreover, Brca1 is highlighted as a molecular “hinge” and as a key component of neuronal differentiation and synaptic coupling [12]. However, the regulatory mechanism of Brca1 expression and the precise roles of Brca1 in CNS development remain unclear.

The mammalian retina is a component of the CNS and consists of six major neuronal cell types forming three layers. These neuronal cells are terminally differentiated and no longer divide. The loss of these cells cannot be reversed and results in partial or permanent functional vision loss. Ischemia, trauma, high intraocular pressure, and inflammation can all result in irreversible DNA damage to the retina, indicated by the DNA ladders observed in DNA gel electrophoresis of damaged retinal neurons [13,14,15] Our previous studies have shown that the expression of Brca1 is involved in DNA homologous recombination (HR) and non-homologues end joining (NHEJ) [5,16]. The efficiency of DNA repair decreases during retina development, as revealed by the end-joining activity assay using nuclear proteins from retina tissue at different developmental stages. Reduced expression of Brca1 by siRNA knockdown significantly inhibits DNA repair and the viability of retina precursor cells. Therefore, Brca1 might be a target for reactivation to enhance retinal neuronal DNA repair.

In addition, Brca1 plays a critical role in the cell cycle, cell differentiation, chromatin remodeling, and transcriptional regulation [8]. For example, a previous study discovered Brca1 to be re-expressed in brain tissue in patients with Alzheimer disease [17]. The authors suggested that Brca1 might modulate neuronal cell cycle re-entry. Moreover, Kondo’s study indicated that Brca1 and the chromatin remodeling protein Brm facilitate the conversion of mouse oligodendrocyte precursor cells into multipotent NSLCs, which can generate both neurons and glial cells [11]. Bromberg et al. predicted the role of Brca1 in neuronal differentiation in silico, generating a signaling network by connecting cannabinoid receptor 1 (CB1R) to 23 activated transcription factors [18]. However, most previous studies investigated the physiological functions of Brca1 using immortal cell lines, which might be different from primary neurons. Thus, the role of Brca1 in primary retinal neurons remains unclear.

To address these questions, we measured Brca1 expression in primary retinal neurons after treating the cells with either the histone deacetylase inhibitor Trichostatin A (TSA) or the DNA methyltransferase inhibitor 5-Aza-CdR and we examined the possible mechanisms influencing Brca1 expression, focusing on the inducibility of Brca1 promoter and on DNA repair. Moreover, we also determined whether transduction of Brca1 affects some properties of primary retinal neurons such as apoptosis, proliferation, and neurite outgrowth. Thus, the current study provides new insights into the mechanisms regulating the transcriptional expression and function of Brca1 in retinal neurons.

## 2. Results

### 2.1. Breast Cancer Type 1 Susceptibility Protein (Brca1) Is Developmentally Downregulated in Rat Retina

**Figure 1:**
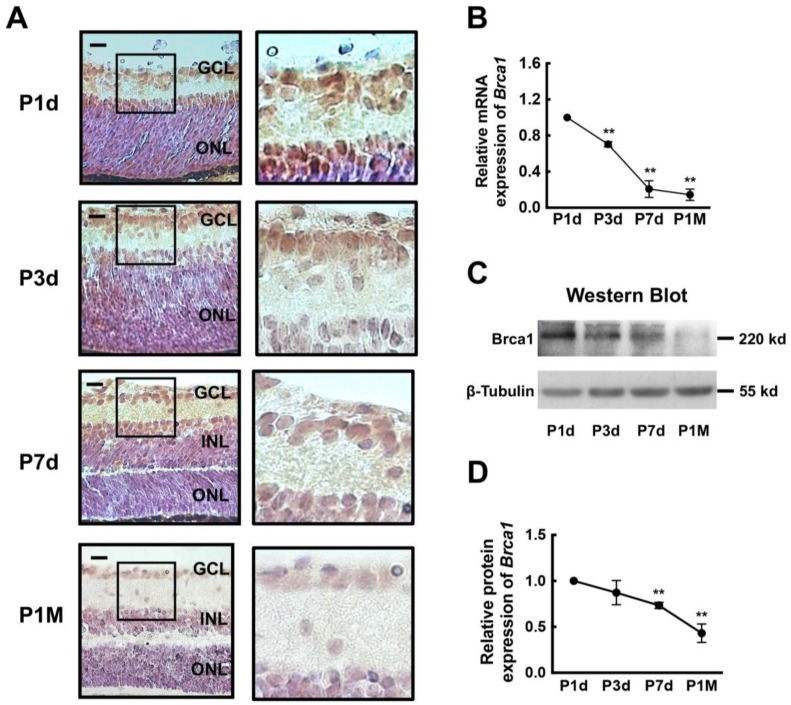
Brca1 is developmentally downregulated in rat retinal neurons. (**A**) Immunohistochemical analysis of Brca1 in postnatal rat retina at different time points. The sections were immunolabeled for Brca1, and the cells’ nuclei were labeled with hematoxylin. Brca1 staining (brown) is intensely detected in the ganglion cell layer (GCL) and in the area close to the GCL of the outer layer in postnatal day 1 (P1d) and postnatal day 3 (P3d) retinas and is detected in the GCL and the inner nuclear layer of postnatal day 7 (P7d) retina. No Brca1 staining is observed in the postnatal month 1 (P1M) retina. Scale bars: 50 μm; (**B**) The mRNA expression level of Brca1 was assayed by real-time reverse transcription-polymerase chain reaction (RT-PCR) and normalized to β-actin levels (** *p* < 0.01). All data were derived from at least three separate experiments; (**C**) The protein expression level of Brca1 was assayed by western blot. β-tubulin was included as a loading control; (**D**) The protein expression level of Brca1 in the retina was quantified by densitometry. Brca1 in the retina significantly and gradually decreases with the age of the rat (** *p* < 0.01). Data are shown as mean ± standard deviation (SD). *n* = 3 and *n* represents separate experiments.

First, an immunohistochemical analysis was performed to measure the expression of Brca1 in the rat retina during development. As shown in Figure 1A, at postnatal day 1 (P1d) and day 3 (P3d), the neural retinas of rodents are composed of two major layers: a large outer layer with neuroblastic cells and an inner layer containing differentiated ganglion cells. A high level of Brca1 immunoreactivity was observed principally in the ganglion cell layer (GCL) and in the area close to GCL of the outer layer. At postnatal day 7 (P7d), there was a distinct three-layer retina. Brca1 was also strongly expressed in the GCL and the inner nuclear layer. Moreover, Brca1 was mainly located in the cell nuclei. At postnatal month 1 (P1M), Brca1 was completely silenced in all three retinal layers. Thus, our data show an age-related pattern of Brca1 expression in the retina. The results of real-time RT-PCR and Western blot assays further confirmed this pattern (Figure 1B,C). The relative expression of Brca1 mRNA was significantly decreased over time in the retinas (P1d, 1.0; P3d, 0.703 ± 0.029; P7d, 0.207 ± 0.092; P1M, 0.144 ± 0.062. ** *p* < 0.01; Figure 1B). Figure 1D shows the relative ratio of Brca1 protein during development. The relative intensities of the bands were quantified by densitometry and normalized to β-tubulin levels. The level of Brca1 protein appeared to significantly decrease as well (P1d, 1.0; P3d, 0.872 ± 0.132; P7d, 0.733 ± 0.034; P1M, 0.431 ± 0.10. * *p* < 0.05, ** *p* < 0.01; Figure 1D). This expression pattern of Brca1 in the retina is consistent with that reported in previous studies, in which Brca1 was silenced in mature neurons in the brain [9,10].

### 2.2. 5-Aza-CdR Upregulates Brca1 Expression in Retinal Neurons

**Figure 2:**
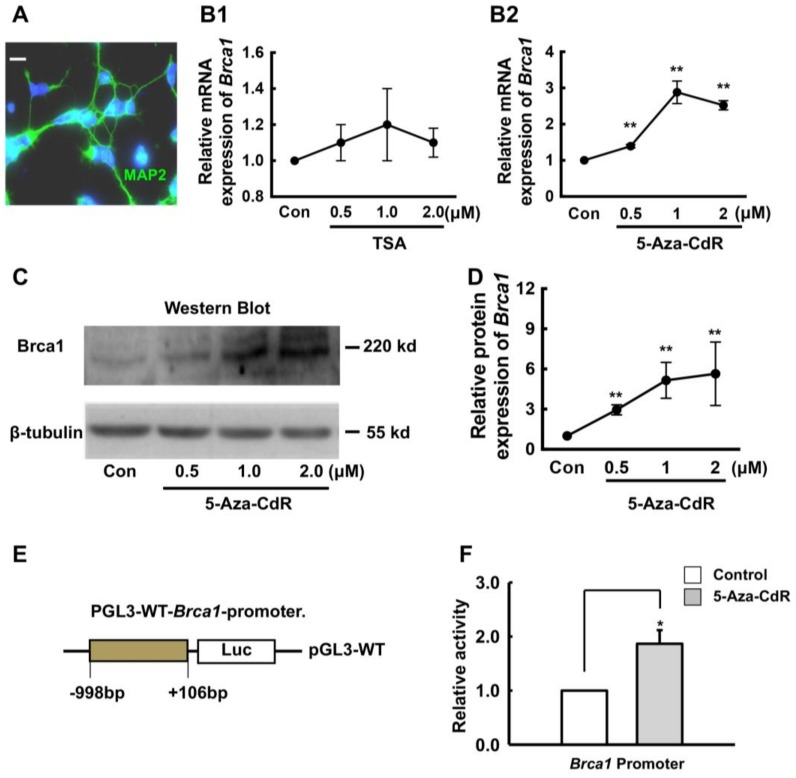
5-Aza-CdR upregulates Brca1 expression in retinal neurons. (**A**) Immunocytochemical staining of MAP2-positive cells (green). Scale bars: 10 μm; (**B**) Primary retinal neurons were treated with different concentrations of 5-Aza-CdR and Trichostatin A (TSA). Real time RT-PCR assays indicate that the mRNA expression level of *Brca1* is upregulated in retinal neurons treated with 5-Aza-CdR (B2) but not with TSA (B1). All data were derived from at least three separate experiments (** *p* < 0.01); (**C**) Western blot analysis of Brca1 protein expression levels indicates a gradual upregulation after 5-Aza treatment. β-tubulin is shown as an internal control; (**D**) The relative quantification of the protein expression of Brca1 in the retina was performed by densitometry (** *p* < 0.01). All data were derived from at least three separate experiments; (**E**) Luciferase plasmid structure; (**F**) The relative activity of the *Brca1* promoter in the retina was quantified by luciferase activity assays. 5-Aza-CdR increases luciferase activity at the *Brca1* promoter (* *p* < 0.05). Data are shown as mean ± SD. *n* = 3 and *n* represents separate experiments.

In order to elucidate the regulatory mechanism of Brca1 in the retina, primary SD P3d rat retinal neurons were treated with 10 μm/mL Ara-C to inhibit cell proliferation on the second day and then were cultured for one week. The cells were then stained with anti-microtubule associated protein 2 (MAP2) antibodies. As shown in Figure 2A, all cells were MAP2-positive (green). Gene silencing is often mediated by histone deacetylation in post-mitotic cells [18]. Thus, the cells were treated with the histone deacetylase inhibitor TSA. Forty-eight hours after treatment, the RNA and total proteins were extracted from neurons. Real-time RT-PCR and western blot were performed to measure Brca1 expression levels. As shown in Figure 2B1, the mRNA level of Brca1 was not changed by histone acetylation.

The DNA methyltransferase inhibitor 5-Aza-CdR is generally thought to act through incorporation into DNA during mitosis, thereby preventing methylation of the new DNA strand. However, 5-Aza-CdR also affects gene expression in post-mitotic, mature neurons [19]. Here, we found that Brca1 mRNA level was increased by 5-Aza-CdR, compared with controls (0 μM, 1.0; 0.5 μM, 1.39 ± 0.047-fold; 1.0 μM, 2.88 ± 0.313-fold; 2.0 μM, 2.52 ± 0.13-fold. ** *p* < 0.01; Figure 2B2). Additionally, this finding was confirmed by western blot analysis (Figure 2C). In fact, Brca1 protein levels were also increased by 5-Aza-CdR in a dose-dependent manner, compared with controls (0 μM, 1.0; 0.5 μM, 2.95 ± 0.37-fold; 1.0 μM, 5.15 ± 1.34-fold; 2.0 μM, 5.64 ± 2.37-fold, respectively. * *p* < 0.05, ** *p* < 0.01; Figure 2D). Furthermore, we analyzed luciferase activity at the *Brca1* promoter following treatment with 5-Aza-CdR. We found that the activity of the *Brca1* promoter was significantly increased in retinal neurons by 1.86-fold by 5-Aza-CdR (* *p* < 0.05, Figure 2F). Thus, 5-Aza-CdR upregulates Brca1 expression in mature retinal neurons.

### 2.3. 5-Aza-CdR Promotes DNA Repair in Retinal Neurons after Ionizing Radiation (IR) Treatment

**Figure 3:**
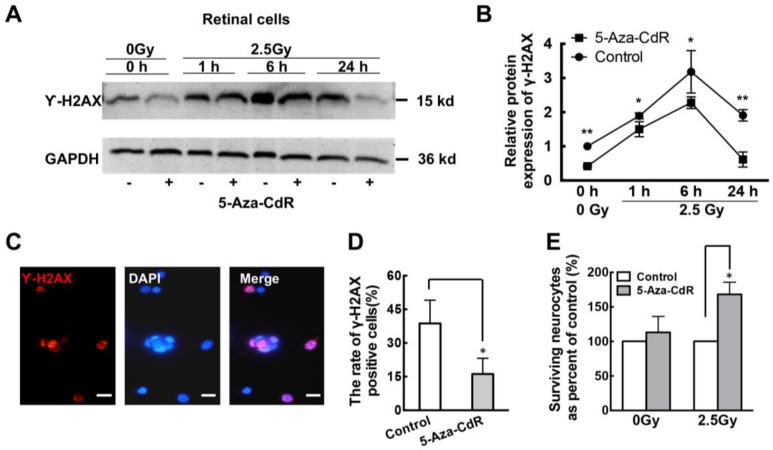
5-Aza-CdR reduces DNA damage of retinal neurons after ionizing radiation (IR) treatment. (**A**) Western blot analysis indicates that phosphorylation of histone H2A.X (γ-H2AX) expression is downregulated in the presence of 5-Aza-CdR in primary retinal neurons undergoing or not IR treatment, compared with neurons that were not exposed to 5-Aza-CdR; (**B**) The relative quantification of the protein expression level of γ-H2AX in the retina was performed by densitometry (** *p* < 0.01; * *p* < 0.05). All data were derived from at least three separate experiments; (**C**) Immunofluorescence staining depicts the spatial localization of phospho-H2AX in the nuclei of primary retinal neurons. Scale bars: 20 μm; (**D**) The number of γ-H2AX positive cells is decreased in the presence of 5-Aza-CdR (* *p* < 0.05). All data were derived from at least three separate experiments; (**E**) The cell viability ratio shows that 5-Aza-CdR significantly increases cell survival in retinal neurons exposed to 2.5Gy IR but not in those undergoing a sham treatment (* *p* < 0.05). Data are shown as mean ± SD. *n* = 3 and *n* represents separate experiments.

Brca1 plays a key role in DNA repair. To further elucidate whether re-expression of Brca1 impacts DNA repair in primary retinal neurons, we investigated the efficiency of DNA damage repair following exposure to IR. After seven days after in culture, SD rat retinal neurons were treated with 1.0 µM 5-Aza-CdR for 24 h before exposure to 2.5Gy IR. A western blot analysis showed that γ-H2AX, a marker of DNA double-strand breaks (DSBs), was significantly decreased by 5-Aza-CdR compared with controls (Figure 3A). A densitometric analysis showed that 5-Aza-CdR treatment significantly decreased γ-H2AX expression, compared to controls without IR treatment (Control, 1.00; 5-Aza-CdR, 0.418 ± 0.029-fold. * *p* < 0.05; Figure 3B). Following exposure to 2.5Gy, a marked decrease in γ-H2AX expression in retinal neurons treated with 5-Aza-CdR was observed during a 24 h period (5-Aza-CdR: 1 h, 1.501 ± 0.221-fold; 6 h, 2.277 ± 0.170-fold; 24 h, 0.615 ± 0.221-fold. Control: 1 h, 1.893 ± 0.084-fold; 6 h, 3.182 ± 0.620-fold; 24 h, 1.909 ± 0.166-fold. * *p* < 0.05, ** *p* < 0.01; Figure 3B). Moreover, the cells were fixed 1 h post-IR and stained for γ-H2AX (Figure 3C). Immunofluorescence analysis showed that the relative ratio of γ-H2AX was decreased by 5-Aza-CdR treatment, compared to controls (Control, 38.7 ± 10.36%; 5-Aza-CdR, 16.17 ± 7.0%. * *p* < 0.05; Figure 3D). In addition, 5-Aza-CdR did not affect the viability of retinal neurons undergoing a sham treatment, but significantly promoted the survival of those exposed to 2.5Gy IR (Figure 3E). Thus, our findings indicate that 5-Aza-CdR treatment enhances genomic stability in retinal neurons, which promotes DNA repair after IR treatment.

### 2.4. Transgenic Brca1 Promotes DNA Repair and Cell Survival of Retinal Neurons after IR Treatment

**Figure 4:**
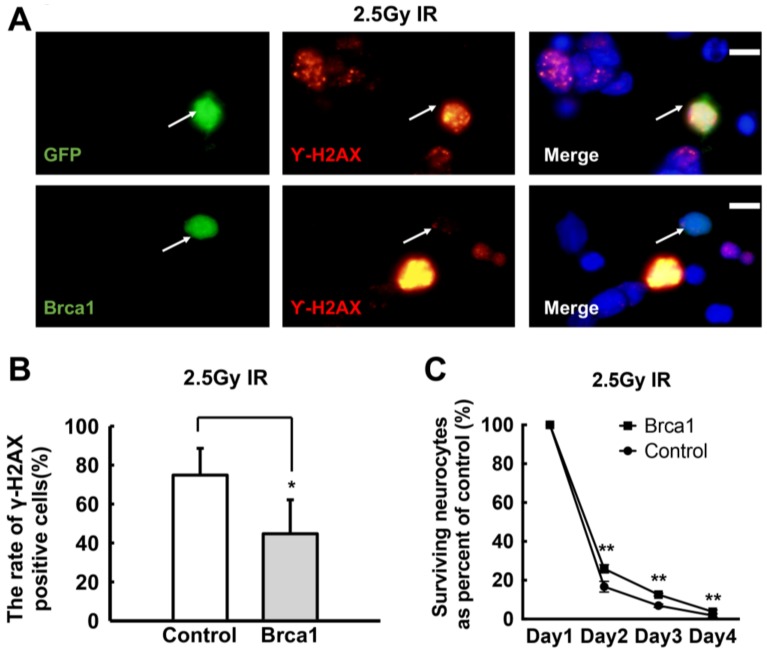
Transgenic Brca1 promotes repair of IR-induced DNA damage and cell survival of retinal neurons. (**A**) Primary retinal neurons were transfected with vector pEPI-eGFP or pEPI-eGFP-Brca1. The cells were exposed to 2.5Gy IR, then fixed and analyzed by immunofluorescence. Immunofluorescence staining images depict the spatial localization of γ-H2AX in the nuclei of primary retinal neurons. The arrows indicate the cells successfully transfected with the plasmids. Scale bars: 10 μm; (**B**) After IR treatment, the percent of γ-H2AX positive cells is decreased in transgenic Brca1-positive retinal cells compared with the vector-positive controls (* *p* < 0.05). All data were derived from at least three separate experiments; (**C**) The cell viability ratio shows that cell survival is increased in Brca1-positive cells, compared to control cells after IR treatment (** *p* < 0.01). Data are shown as mean ± SD. *n* = 3 and *n* represents separate experiments.

5-Aza-CdR induces the re-expression of Brca1 in retinal cells. However, 5-Aza-CdR might induce global changes in the cells, which affects a massive number of genes. Thus, to evaluate the effects of Brca1 on retinal neurons more precisely, exogenous Brca1 fused to GFP was transfected into the cells. Brca1 is involved in DNA repair, so immunostaining of γ-H2AX was performed in retinal neurons transfected with a sham vector or with pEPI-eGFP-Brca1. Forty-eight hours after transfection, the cells were exposed to 2.5Gy IR or to a sham treatment. The cells were fixed 1 h post-damage and stained with anti-γ-H2AX antibodies. Co-localization of GFP and γ-H2AX was observed both in cells transfected with the sham vector and in those transfected with Brca1 (Figure 4A). When the cells were not treated with IR, no difference in the relative number of γ-H2AX-positive cells was observed between vector (GFP)-expressing and exogenous Brca1-expressing cells (data not shown). However, after IR treatment, the relative number of γ-H2AX-positive cells was significantly lower in Brca1-positive cells (44.74 ± 17.4%), compared with sham vector-transfected cells (74.88 ± 13.73%) (* *p* < 0.05; Figure 4B). Moreover, cell survival was increased in transgenic Brca1-positive cells, compared with sham vector-transfected cells after IR treatment. (Control: day 2, 16.67 ± 2.79%; day 3, 6.91 ± 0.83%; day 4, 183 ± 0.88%; Brca1: day 2, 25.92 ± 2.11%; day 3, 12.58 ± 0.68%; day 4, 3.75 ± 0.96%; respectively, ** *p* < 0.01; Figure 4C). Thus, these results indicate that transduction of Brca1 promotes DNA repair and improves cell survival in retinal neurons after IR treatment.

### 2.5. Transgenic Brca1 Inhibits Neurite Outgrowth in Retinal Neurons

**Figure 5:**
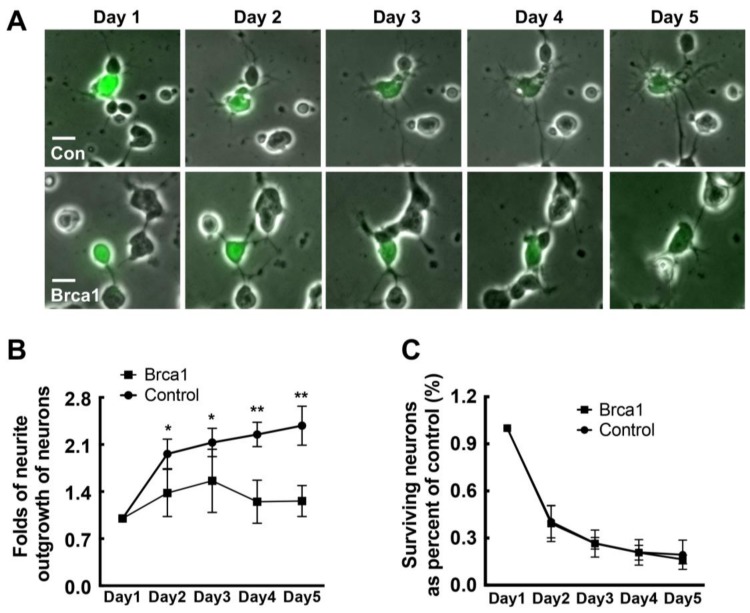
Transgenic Brca1 inhibits neurite outgrowth in retinal neurons. (**A**) Primary retinal neurons were transfected with either vector pEPI-eGFP or pEPI-eGFP-Brca1. Scale bars: 20 μm; (**B**) The mean outgrowth of neurites in Brca1-GFP-positive cells is significantly decreased compared to the GFP-positive cells from day 2 to day 5 (** *p* < 0.01; * *p* < 0.05). All data were derived from at least three separate experiments; (**C**) The cell viability ratio shows no significant difference between cells transfected with the empty vector and those transfected with pEPI-eGFP-Brca1. Data are shown as mean ± SD. *n* = 3 and *n* represents separate experiments.

Brca1 might play a critical role in the cell cycle, cell differentiation, etc. Hence, neurite outgrowth was examined in neurons transfected with pEPI-eGFP or pEPI-eGFP-Brca1 (Figure 5A). At different time points after transfection, we measured neurite outgrowth in GFP-positive cells. We analyzed more than 100 GFP-positive cells at different time points and measured the length of each neurite. As shown in Figure 5B, no difference in neurite outgrowth was observed between cells expressing exogenous Brca1 and those transfected with the empty vector at day 1 post-transfection. However, from the second to fifth day, neurite outgrowth in neurons transfected with pEPI-eGFP-Brca1 was significantly decreased compared to controls transfected with the empty vector pEPI-eGFP (Control: day 2, 1.96 ± 0.22-fold; day 3, 2.13 ± 0.21-fold; day 4, 2.25 ± 0.18-fold; day 5, 2.38 ± 0.28-fold; Brca1: day 2, 1.37 ± 0.35-fold; day 3, 1.56 ± 0.47-fold; day 4, 1.24 ± 0.32-fold; day 5, 1.26 ± 0.23-fold; * *p* < 0.05, ** *p* < 0.01; Figure 5B). Thus, these results indicate that Brca1 might prevent neural differentiation and affect cell morphology.

Since Brca1 might be involved in conversion in neurons, we analyzed proteins related to cell apoptosis and reprogramming (Caspase-3,5-ethynyl-2′-deoxyuridine (EdU), Nestin, etc.) in single retinal neurons expressing exogenous Brca1. Our data show that transduction of Brca1 produced no changes in proliferation or reprogramming in retinal neurons (Figures S1 and S2). Moreover, transgenic Brca1 did not affect cell viability during the 5-days period (*p* > 0.05; Figure 5C).

## 3. Discussion

In the present study, we show that Brca1 is downregulated during rat retina development. Moreover, we found that 5-Aza-CdR treatment induces Brca1 re-expression in primary retinal neurons and alleviates DNA damage following IR exposure. Furthermore, transduction of Brca1 also promotes DNA repair and cell survival of IR-treated retinal neurons. Therefore, our data indicate that Brca1 promotes genomic stability and improves cell viability of retinal neurons after IR treatment. In addition, Brca1 may prevent cell differentiation, as neurite outgrowth of retinal neurons expressing exogenous Brca1 was significantly inhibited compared with controls. Thus, our present findings provide direct biochemical and functional evidence supporting a function of Brca1 in retinal neuronal development.

### 3.1. Expression Pattern of Brca1 in Retinal Development

Our data show that Brca1 is highly expressed in the rat retina early after birth and gradually decreases with age, eventually becoming silenced by P1M. Moreover, Brca1 is mainly expressed in the GCL and the inner nuclear layer of the retina at postnatal day 1. These results are consistent with previous reports investigating brain development. Brca1 is only expressed in proliferating zones of the fetal hippocampus [12] and in immature neural cells in the walls of brain ventricles and in the choroid plexus of the adult brain, which consist of neural stem cells [9,10]. We also performed double immunostainings to identify Brca1 expression in ganglion cells, astrocytes, Müller cells in the retina. Figure S3 shows that Brca1 is expressed in both the nuclei and the cytoplasm in Thy1.1-positive ganglion cells, GFAP-positive astrocytes, and GS-positive Müller cells in vitro. Therefore, these results suggest that the retinal neurons at P1d are immature cells.

Furthermore, our data demonstrate that 5-Aza-CdR significantly upregulates Brca1 expression in retinal neurons in vitro. The transcriptional activity of the *Brca1* promoter was markedly increased in retinal neurons following 5-Aza-CdR treatment. 5-Aza-CdR is generally thought to incorporate into DNA during mitosis, thereby preventing methylation of the new DNA strand. However, since primary retinal neurons are non-dividing cells, 5-Aza-CdR could not activate Brca1 expression by demethylation as usual, which is also supported by previous studies. Aldiri et al. profiled the epigenetic and transcriptional changes that occur during retinogenesis and reprograming. They observed that Brca1 is silenced in retinal mature neurons and found no methylated sites in *Brca1* promoter [20]. In addition, 5-Aza-CdR can also bind RNA. 5-Aza-CdR could affect gene expression through its incorporation into DNA and RNA [21,22]. Moreover, Vitalina’s study also demonstrated that 5-Aza-CdR treatment reorganizes genomic histone modification patterns. Most changes in gene expression are not due to the relief of repression mediated by DNA or histone methylation [23]. Hence, further research is needed to elucidate the upstream regulation of Brca1 expression.

### 3.2. Brca1 Promotes DNA Repair and Cell Viability of Retinal Neurons

The roles of Brca1 are well characterized in proliferating cells [24] but not in non-dividing neurons. Here, we also demonstrate that Brca1 reduces spontaneous DNA double-strand breaks, as revealed by the decrease in γ-H2AX levels in retinal neurons treated with 5-Aza-CdR (Figure 3). Transduction of Brca1 can significantly promote DNA repair in retinal neurons treated with IR (Figure 4). This is consistent with Shapiro’s report that ethanol-treated *Brca1*-deficient embryos exhibit much higher γ-H2AX levels than ethanol-treated wild-type *Brca1* littermates [25]. Our previous report showed that Brca1 plays an important role in DNA repair and cell viability in retinal cells [16]. Therefore, our data strongly support that Brca1 participates in DNA repair in retinal neurons.

Moreover, Brca1 did not affect the viability of normally cultured retinal neurons (Figure 5C), whereas, significantly promotes cell survival after IR treatment (Figure 4C). These results imply that Brca1 increases cell viability by upregulation of the DNA double-strand breaks repair in retinal neurons. A previous study reported that 5-Aza-CdR contributes to delaying ischemic brain injury. It suggested that 5-aza-dC could be incorporated into newly repaired DNA of affected neurons because different DNA repair pathways occur in mature neuron after ischemia–reperfusion or IR treatment [26,27,28,29]. Therefore, we speculate that Brca1 upregulation by 5-Aza-CdR also plays an important role in neural protection after ischemic damage.

### 3.3. Brca1 Silencing Might Promote Cell Maturation of Retinal Neurons

More interestingly, our data show that transgenic Brca1 significantly inhibits neurite outgrowth of retinal neurons (Figure 5). Therefore, Brca1 silencing during development (Figure 1) might induce neurite outgrowth, which might be involved in cell movement and the formation of three retinal layers after birth. This hypothesis is supported by previous studies. Aldiri’s study suggested that most downregulated genes encode proteins contributing to neuronal differentiation in the developing mouse retina [20]. Loss of BRCA1 expression by siRNA interference promotes neurite outgrowth and increases the synaptic density of neural-like cells (Neuro2A) [18]. To identify the upstream signaling pathways and components regulating the activated transcription factors the authors constructed a network in silico, using available protein–protein interaction databases, graph-theory analysis, and statistical tests. BRCA1 was identified as a specific interactor in activated transcription. At least 23 transcription factors are regulated by Brca1, such as, PI3K, PAX6, and CB1R. Therefore, the developmental silencing of Brca1 might play a key role in differentiation and determination of neuronal morphology.

In addition, Brca1 participates in gene transcription, apoptosis, cell proliferation, and cell differentiation in proliferating cells [30]. Brca1 is expressed in neural stem cells and has been identified as a “hinge” in the development of CNS [18]. Therefore, we analyzed proteins related to cell apoptosis and reprogramming (Caspase-3, EdU, Nestin, etc.) in single retinal neurons expressing exogenous Brca1. However, our data showed that transduction of Brca1 does not change the expression of these markers (propidium iodide (PI), Caspase-3, EdU, Nestin) in retinal neurons compared with the mock vehicle group (Figures S1 and S2). Kondo et al. also reported that Brca1 reactivates Sox2 promoter by interacting with the chromatin-remodeling protein Brm, which induces oligodendrocyte precursor cells to convert to multipotent neural stem-like cells [11]. Therefore, the activity of Brca1 in neuron reprograming might depend on the cooperation with other factors, a hypothesis that will be investigated in our future study.

## 4. Materials and Methods

### 4.1. Ethics Statement

All the animal studies were performed at the animal facilities of the Ophthalmic Animal Laboratory, Zhongshan Ophthalmic Center, Sun Yat-sen University, in accordance with the guidelines approved by the Institutional Animal Care and Use Committee of Zhongshan Ophthalmic Center (Permit Number: SYXK (YUE) 2010-0058, 30 May 2015). The animals were raised at the suitable temperature (16–26 °C) and relative humidity (40–70%) and on a light schedule of 12 h–12 h light–dark cycle (lights on at 7:00 a.m., 200 lux). Rats were housed four per cage and maintained with continuous access to food and water. Animal health was monitored daily by the animal care staff and veterinary personnel. SD rats were sacrificed by an intraperitoneal injection of 10% chloral hydrate (0.4 mL/100 g) (Sigma-Aldrich, St. Louis, MO, USA) to minimize suffering before we harvested the retinas.

### 4.2. Immunohistochemistry

Rat eye sections at postnatal day 1, day 3, day 7, and 1 month were prepared. We performed the IHC staining according to the manufacturer’s protocols (SABC-AP kit; Boster, Wuhan, China). The sections were fixed in 4% paraformaldehyde, then immersed in hydrogen peroxide in methanol (30% hydrogen peroxide:methanol = 1:50) to inactivate endogenous peroxidase. The slides were blocked with 5% BSA and incubated at 4 °C overnight with the primary antibody, anti-Brca1 antibody (20649-1-AP, Proteintech, Wuhan, China). The biotin-labeled goat anti-rabbit IgG antibody was used as secondary antibody. The immunostaining was visualized with DAB (Boster, Wuhan, China) treatment. Finally, the sections were counterstained with hematoxylin and mounted.

### 4.3. Primary Sprague-Dawley (SD) Rat Retinal Neurons Culture and Treatment

Postnatal 1-day-old SD rats were provided by the animal center of Sun Yat-sen University, Guangzhou, China. Their retinas were dissociated by trypsin to gain a suspension of single cells. Briefly, postnatal 1-day-old SD rat were sacrificed by an intraperitoneal injection of 10% chloral hydrate (0.4 mL/100 g) (Sigma-Aldrich, St. Louis, MO, USA). The retinas, separated from the eyeballs, were incubated for 15 min at 37 °C in a solution containing 0.125% trypsin with ethylenediaminetetraacetic acid (EDTA) in order to dissociate the cells. After trypsinization was terminated by addition of Dulbecco’s modified eagle medium (Gibco, Carlsbad, CA, USA) supplemented with 10% fetal bovine serum (Gibco, Carlsbad, CA, USA), the cells were passed through a 70 μm cell strainer and collected by centrifugation for 5 min at 1000 rpm. The cell density was adjusted to approximately 1 × 10^6^ cells/mL. Then, the cells were seeded on culture plates pre-coated with 0.01% poly-d-lysine (Sigma-Aldrich, St. Louis, MO, USA) and incubated at 37 °C in an atmosphere containing 5% CO_2_ and 95% air. After cultured for 12 h, the cells were treated with 10 μm/mL Ara-C (Sigma-Aldrich, St. Louis, MO, USA) to suppress the growth of non-neurons. After 12 h, the medium was substituted with a complete medium (10% fetal bovine serum). MAP2 (Boster, Wuhan, China) staining was performed to determine the character of the cultured cells.

To upregulate Brca1 expression, the cells were treated with either a methyltransferase inhibitor, 5-Aza-CdR (Sigma-Aldrich, St. Louis, MO, USA), or a HDAC inhibitor, Trichostatin A (TSA) (Sigma-Aldrich, St. Louis, MO, USA). The control cells were treated in parallel with the corresponding solvent (dimethyl sulfoxide and phosphate buffer saline (PBS)).

### 4.4. Real-Time PCR

Total RNA from retina of postnatal days 1, 3, 7 rats and 1-month-old SD rats were isolated with Trizol Reagent (Invitrogen, Carlsbad, CA, USA). One microgram of total RNA was subjected to reverse transcription using a cDNA synthesis kit (Takara, Beijing, China) following the manufacturer’s protocol. The following primers were used: Brca1 (sense 5′-TGTCCTTCATGCTATGCAGA-3′; antisense 5′-GCACTTCCTTGTAGGCTCCT-3′) and β-actin (sense 5′-TCAGGTCATCACTATCGGCAAT-3′; antisense 5′-AAAGAAAGGGTGTAAAACGCA-3′). The expression levels of Brca1 and β-actin were measured by real-time PCR, using the Roche 480 System (Roche, Indianapolis, IN, USA).

### 4.5. Western Blot

Western blotting was carried out by standard protocols. Anti-Brca1 (sc-28234, Santa Cruz, CA, USA) and anti-Phospho-Histone H2A.X (Ser139) (2577S, Cell Signaling Technology, Beverly, MA, USA) were used as primary antibodies. GAPDH (10494-1-AP, Proteintech, Chicago, IL, USA) and ß-Tublin (sc-9104, Santa Cruz, CA, USA) served as loading controls. The protein bands were detected using an Enhanced Chemiluminescence Detection System (Millipore, Billerica, MA, USA). Each experiment was repeated at least three times.

### 4.6. Luciferase Activity Assays

The *Brca1* promoter region (−998 to +106) in PGL3-WT was generated as follows. The required *Brca1* promoter region was amplified by PCR using the primer pair: (5′-GGGGTACCCCGACAGAGACCGGGCCTAGTC-3′ and 5′-CCCAAGCTTGGGTGTCTCACCTTTCTTCCGAG -3′) (Kpn I and Hind III sites were used, respectively). The PCR products and vector PGL3-WT were digested with Kpn I and Hind III, and the *Brca1* promoter fragment (1105 bp) was inserted into PGL3-WT at the corresponding sites following the manufacturer’s protocol, yielding the construct PGL3-WT-Brca1-promoter. The integrity of the fragment was verified by DNA sequencing.

The luciferase assays were performed to examine the activity of the *Brca1* promoter region. Primary rat retinal neurons were transfected using Lipofectamine 2000 (Invitrogen, Carlsbad, CA, USA) as previously described. The transfected plasmid mixture included 2 μg of different reporter plasmids and 2 μg of expression plasmids or pcDNA3-based vectors. The pCMV-RL plasmid (Promega, Madison, WI, USA) encoding Renilla luciferase was used to monitor the transfection efficiency in each sample. After 24 h, the levels of firefly and Renilla luciferase activity were detected sequentially by a Dual-Glo Luciferase Assay Kit (Promega, Madison, WI, USA). The levels of firefly luciferase activity were analyzed after normalization to Renilla luciferase activity.

### 4.7. Plasmid Construction and Transfection

The plasmid pEPI-GFP-Brca1 was derived from pEPI-GFP [31], by inserting rat Brca1 cDNA into pEPI-GFP after restriction digestion at SacII and XmaI sites (New England Biolabs, Beverly, MA, USA). The integrity of the fragment was verified by DNA sequencing.

The plasmid pEPI-EGFP-Brca1 was transfected into the SD rat primary retinal neurons. The transfection was performed according to the protocol provided by the transfection kit (Amaxa™ Basic Nucleofector™ Kit, Gaithersburg, MD, USA) (program: G-013) and utilizing the Amaxa Nucleofector II device (Lonza, Rockland, ME, USA). For each transfection, 12 μg of plasmid DNA pEPI-EGFP-Brca1 or empty vector) was used. After 24–120 h of incubation, the cells were recorded by live cell imaging. Some cells were exposed to 2.5Gy IR or a sham treatment, then fixed and analyzed by immunofluorescence.

### 4.8. Assay of Retinal Neurite Outgrowth, Cell Mortality, Cell Differentiation, Cell Proliferation, and DNA Stability

The cells were transfected with the target plasmids and recorded by live cell imaging for 120 h. The mortality ratio of the target cells was determined by the number of daily dead cells divided by the total number of cells. A camera lucida projection onto concentric circles was used to assess the neurite outgrowth of retinal neurons. The outgrowth was calculated by summing the growth lengths of all the cells and dividing by the total number of cells in 50× microscopic fields. The average mortality ratio and relative percent of outgrowth of retinal neurons (at least *n* = 3 for each experimental condition) is reported in the text and figures as the mean ± 2 SD.

Immunofluorescence assays of PI (Boster, Wuhan, China) and anti-Caspase3 (#9664, Cell Signaling Technology, Beverly, MA, USA) were performed to assay the viability of cells which were successfully transfected with the plasmids. Double immunofluorescence staining of Nestin (ab82375, Abcam, Cambridge, MA, USA) and MAP2 (BM1243, Boster, Wuhan, China) were performed to measure cell differentiation. An EdU cell proliferation test kit (RiboBio, Guangzhou, China) was used to measure cell proliferation. The cells, after transfection with the target plasmids, were cultured for 48 h and then exposed to 5 μmol/L of 5-ethynyl-20-deoxyuridine for additional 4 h at 37 °C. The cells were fixed with 4% paraformaldehyde for 15 min and incubated in 2 mg/mL glycine for 5 min at room temperature. After treatment with 0.5% Triton X-100 for 10 min and washing with PBS three times, the cells in each well were incubated with 100 μL of 1× Apollo^®^ reagent for 30 min. Then, 100 μL of Hoechst 33342 (5 μg/mL) was added and left in each well for 30 min to stain the DNA, and then the cells were observed under a fluorescent microscope.

To assay the DNA stability in retinal neurons after IR treatment, the cells were stained with Phospho-Histone H2A.X (Ser139) (2577S, Cell Signaling Technology, Beverly, MA, USA). Images were obtained by a fluorescence microscope equipped with a camera (Carl Zeiss, MicroImaging GmbH, Göttingen, Germany). The amount of γ-H2AX-positive foci was counted in at least 100 cells and was scored in images obtained using the same exposure time.

### 4.9. Statistical Analysis

The data shown are representative of three independent experiments. The data are expressed in the form of mean ± standard deviation. Student’s *t*-test were used to evaluate the statistical comparisons between two groups and 1-way ANOVA with the least significant difference (LSD) multiple comparison test to evaluate the statistical comparisons between several groups. SPSS for Windows Version 10.5 software package was used to perform statistical analyses (SPSS, Chicago, IL, USA); *p* values less than 0.05 were considered to be statistically significant.

## 5. Conclusions

In conclusion, this study demonstrates that 5-Aza-CdR upregulates Brca1 in retinal mature neurons. Both 5-Aza-CdR and transgenic Brca1 can increase cell viability and promote DNA DSBs repair in retinal neurons after IR treatment. Moreover, Brca1 silencing might promote cell differentiation and play a key role in determination of neuronal morphology. Therefore, the present study provides biochemical and histochemical evidence supporting that Brca1 could be a target for neuroprotection under damage conditions and 5-Aza-CdR a potential drug for the therapy of retinal diseases.

## Supplementary Materials

Supplementary materials are available online at http://www.mdpi.com/1422-0067/19/4/1214/s1.

## Abbreviations

Brca1	Breast cancer type 1 susceptibility protein
CNS	Central nervous system
NSLC	Neural stem-like cell
NHEJ	Non-homologues end joining
TSA	Trichostatin A
IR	Ionizing Radiation
PI	Propidium iodide
EdU	5-ethynyl-2′-deoxyuridine
GCL	Ganglion cell layer
