# Supplementary material for: Brca1 Is Upregulated by 5-Aza-CdR and Promotes DNA Repair and Cell Survival, and Inhibits Neurite Outgrowth in Rat Retinal Neurons
Source: Int J Mol Sci. 2018 Apr 17;19(4):1214. doi: 10.3390/ijms19041214 (PMC5979323; doi:10.3390/ijms19041214)
Supplement: Supplementary file 1 [file ijms-19-01214-s001.zip › Supplementary File1.pdf]

Figure S1

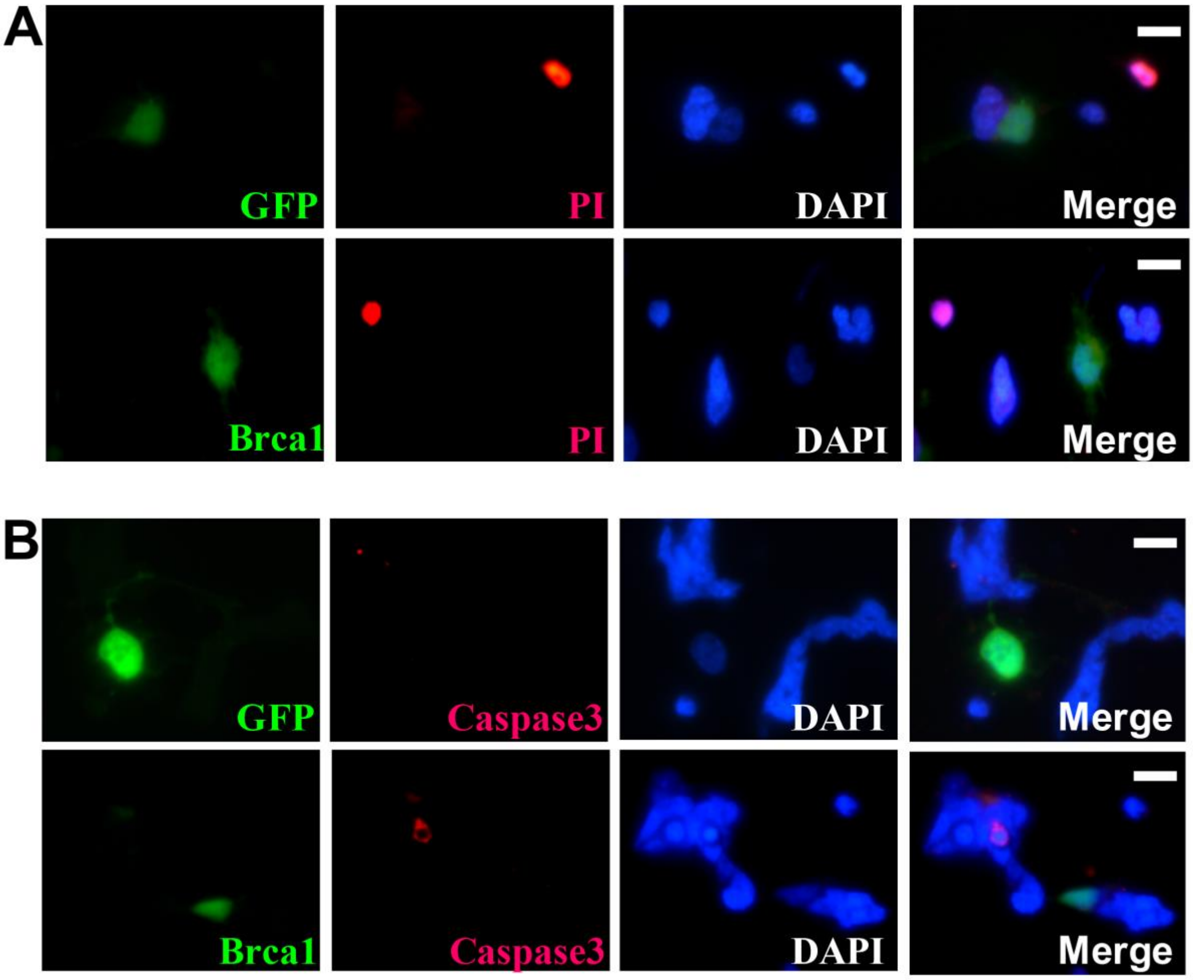

Figure S1. Individual exogenous Brca1 does not affect cell apoptosis in retinal neurons. The primary retinal neurons were transfected with vector pEPI-eGFP-N1 or pEPI-eGFP-Brca1. After 48 hours, cells were processed for immunofluorescence staining for PI (A) and Caspase3 (B). Immunofluorescence staining images indicate that GFP and Brca1-positive cells are both absent for PI and Caspase3. Scale bars: 20 μm. (All data come from at least three separate experiments).

Figure S2

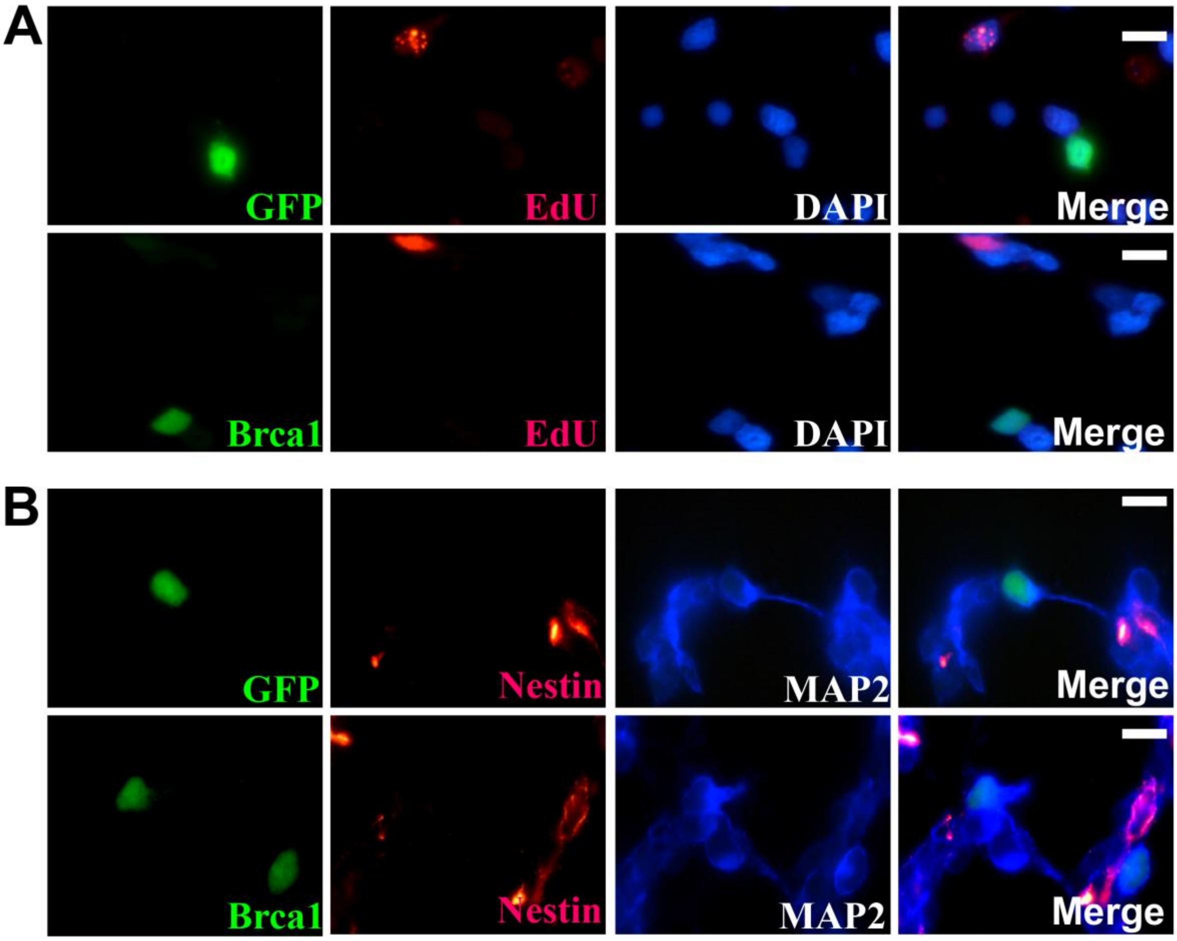

Figure S2. Individual exogenous Brca1 does not affect cell proliferation in retinal neurons. Primary retinal neurons were transfected with vector pEPI-eGFP-N1 or pEPI-eGFP -Brca1. **A.** After 24 hours, cells were treated with 10μM EdU. 24 hours later after treatment, cells were fixed and stained with Apollo solution to detect EdU. EdU is not observed in Brca1-positive cells. **B.** Nestin-staining shows that neither GFP-positive cells nor Brca1-positive cells expresses Nestin. Scale bars: 20 μm. (All data come from at least three separate experiments).

Figure S3

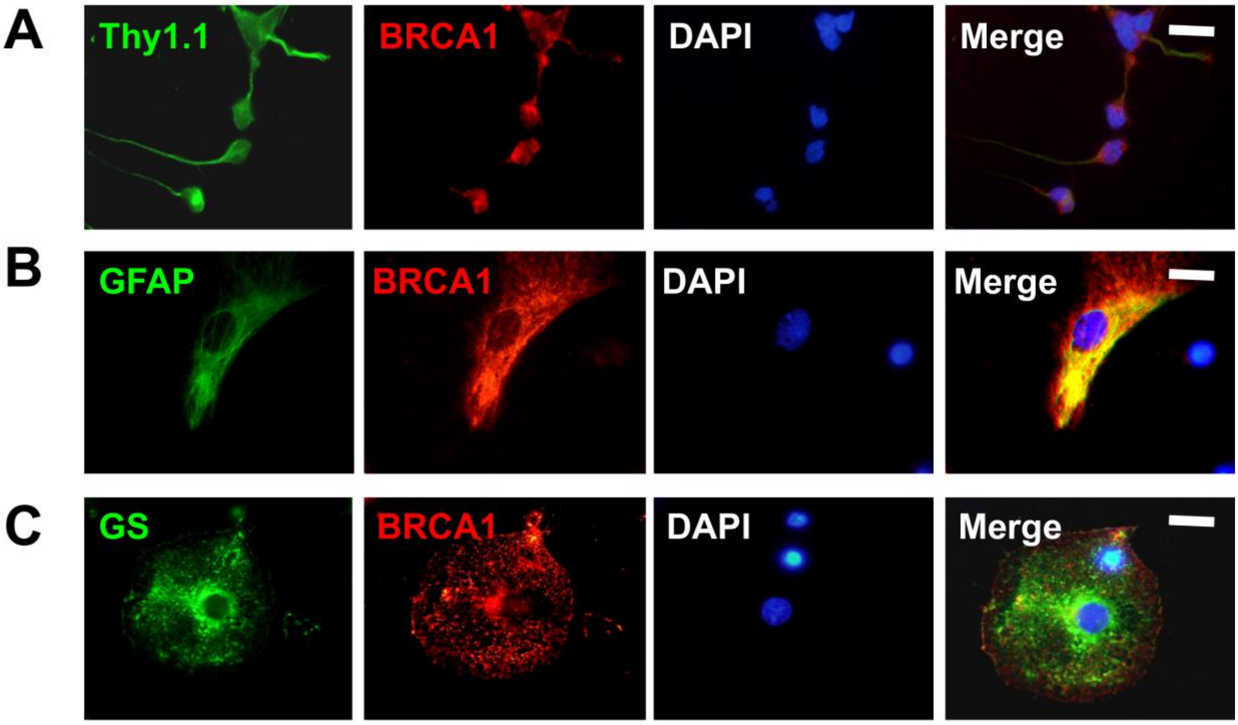

Figure S3. Double staining was performed in SD postnatal 1-day retinal neurons. The nuclei of the cells were labeled with DAPI (blue). Brca1 (red) and Thy1.1 (A), GFAP (B), GS (C) (green). Double-staining shows that in the primary retinal cells, Brca1 is detected both in the cytoplasm and nuclei of Thy1.1-positive neurons, and detected in GS-positive müller cells and GFAP-positive glia cells. Scale bars: 20 μm. (All data come from at least three separate experiments).
