# Supplementary figures and images for: Brca1 Is Upregulated by 5-Aza-CdR and Promotes DNA Repair and Cell Survival, and Inhibits Neurite Outgrowth in Rat Retinal Neurons
Source: Int J Mol Sci. 2018 Apr 17;19(4):1214. doi: 10.3390/ijms19041214 (PMC5979323; doi:10.3390/ijms19041214)

**Figure S1**

**Figure 1C**

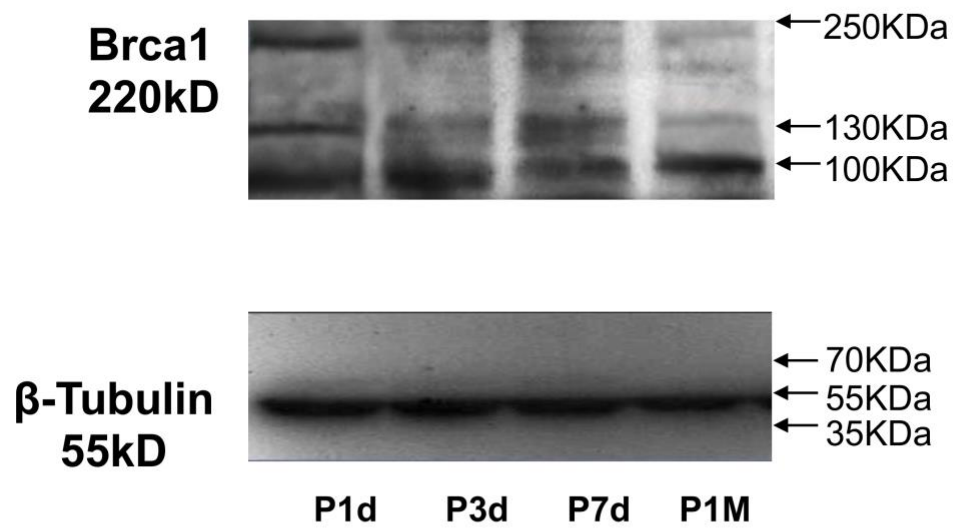

**Figure S2**

**Figure 2C**

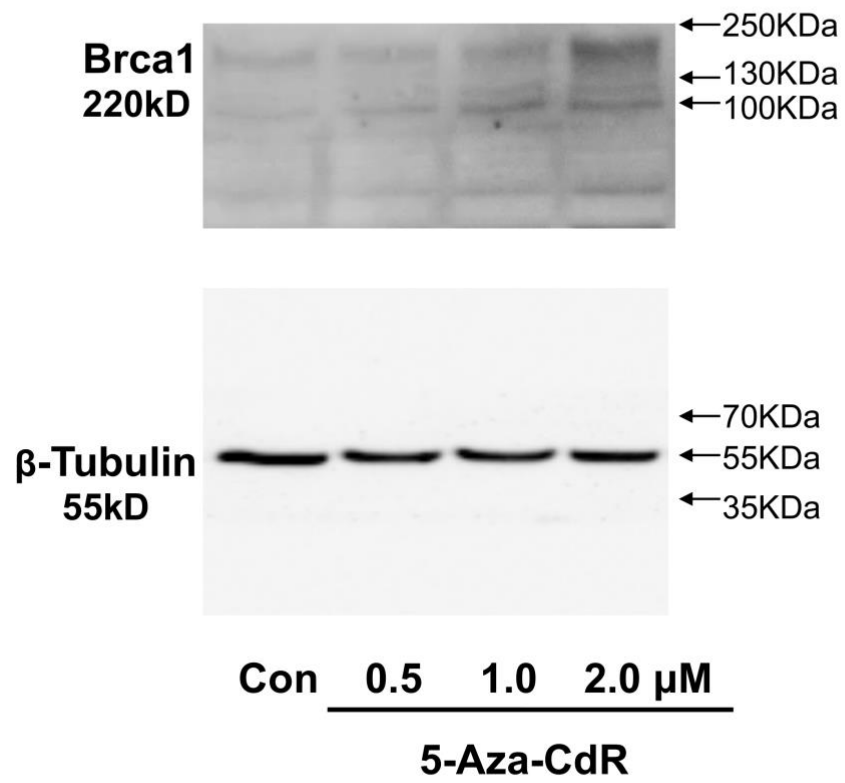

Figure S3

Figure 3A

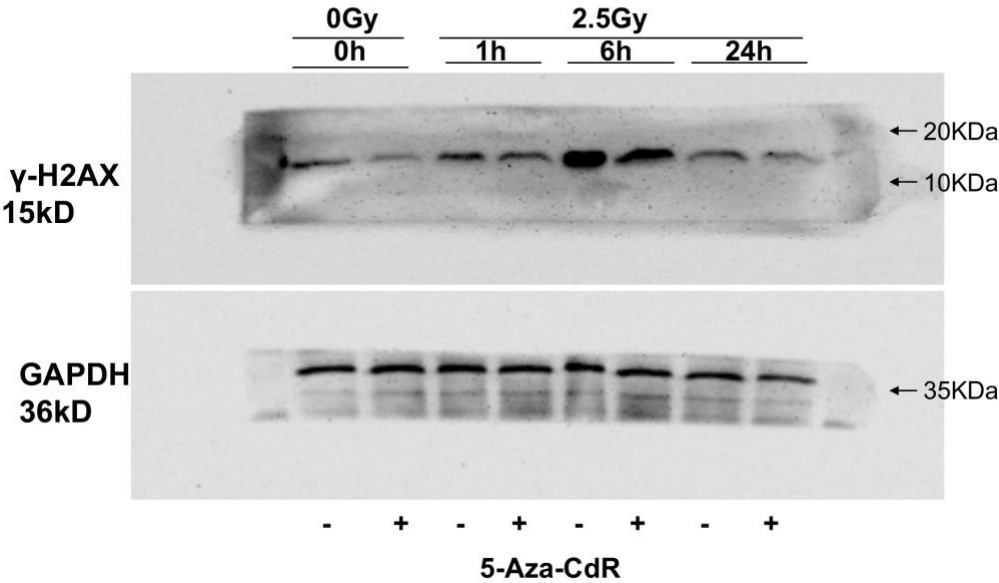

Supplement: Supplementary file 1 [file ijms-19-01214-s001.zip › Supplementary File2.pdf]
